# Integrated analysis of human genetic association study and mouse transcriptome suggests *LBH* and *SHF* genes as novel susceptible genes for amyloid-β accumulation in Alzheimer’s disease

**DOI:** 10.1007/s00439-018-1906-z

**Published:** 2018-07-13

**Authors:** Yumi Yamaguchi-Kabata, Takashi Morihara, Tomoyuki Ohara, Toshiharu Ninomiya, Atsushi Takahashi, Hiroyasu Akatsu, Yoshio Hashizume, Noriyuki Hayashi, Daichi Shigemizu, Keith A. Boroevich, Manabu Ikeda, Michiaki Kubo, Masatoshi Takeda, Tatsuhiko Tsunoda

**Affiliations:** 1 Laboratory for Medical Science Mathematics, RIKEN Center for Integrative Medical Sciences, 1-7-22 Suehiro-cho, Tsurumi-ku, Yokohama, 230-0045 Japan; 2 Tohoku Medical Megabank Organization, Tohoku University, 2-1, Seiryo-machi, Aoba-ku, Sendai, 980-8573 Japan; 3 Department of Psychiatry, Graduate School of Medicine, Osaka University, Osaka, 565-0871 Japan; 4 Department of Neuropsychiatry, Graduate School of Medical Sciences, Kyushu University, 3-1-1 Maidashi, Higashi-ku, Fukuoka, 812-8582 Japan; 5 Department of Epidemiology and Public Health, Graduate School of Medical Sciences, Kyushu University, 3-1-1 Maidashi, Higashi-ku, Fukuoka, 812-8582 Japan; 6 Laboratory for Statistical Analysis, RIKEN Center for Integrative Medical Sciences, Yokohama, 230-0045 Japan; 7 Department of Genomic Medicine, Research Institute, National Cerebral and Cardiovascular Center, Osaka, 565-8565 Japan; 8 Graduate School of Medical Sciences and Medical School, Nagoya City University, Nagoya, 467-8601 Japan; 9 Institute of Neuropathology, Fukushimura Hospital, Toyohashi-shi, Aichi, 441-8124 Japan; 10 RIKEN Center for Integrative Medical Sciences, 1-7-22 Suehiro-cho, Tsurumi-ku, Yokohama, 230-0045 Japan; 11 Department of Medical Science Mathematics, Medical Research Institute, Tokyo Medical and Dental University, 1-5-45 Yushima, Bunkyo-ku, Tokyo, 113-8510 Japan; 12 Present Address: Division of Genomic Medicine, Medical Genome Center, National Center for Geriastrics and Gerontology, 7-430 Morioka-cho, Obu, Aichi 474-8511 Japan

## Abstract

Alzheimer’s disease (AD) is a common neurological disease that causes dementia in humans. Although the reports of associated pathological genes have been increasing, the molecular mechanism leading to the accumulation of amyloid-β (Aβ) in human brain is still not well understood. To identify novel genes that cause accumulation of Aβ in AD patients, we conducted an integrative analysis by combining a human genetic association study and transcriptome analysis in mouse brain. First, we examined genome-wide gene expression levels in the hippocampus, comparing them to amyloid Aβ level in mice with mixed genetic backgrounds. Next, based on a GWAS statistics obtained by a previous study with human AD subjects, we obtained gene-based statistics from the SNP-based statistics. We combined *p* values from the two types of analysis across orthologous gene pairs in human and mouse into one *p* value for each gene to evaluate AD susceptibility. As a result, we found five genes with significant *p* values in this integrated analysis among the 373 genes analyzed. We also examined the gene expression level of these five genes in the hippocampus of independent human AD cases and control subjects. Two genes, *LBH* and *SHF*, showed lower expression levels in AD cases than control subjects. This is consistent with the gene expression levels of both the genes in mouse which were negatively correlated with Aβ accumulation. These results, obtained from the integrative approach, suggest that *LBH* and *SHF* are associated with the AD pathogenesis.

**Electronic supplementary material:**

The online version of this article (10.1007/s00439-018-1906-z) contains supplementary material, which is available to authorized users.

## Introduction

To identify genes affecting phenotypes including diseases, animal models are very useful. Experimental studies in animal models (e.g., mouse) have an advantage in identifying phenotype-related genes and clarifying their functional roles because experiments can be done with intervention controlling for genetic background, age and environments of the animals. There are several approaches for clarifying phenotypic effects of genes (transgenic or knock-out animals, mutagenesis with ENU, RNAi experiment, transcriptome, etc.) (Gondo 2008). Many types of animal models for human disease were constructed to examine the functional roles of genes. A limitation of this approach is that it is usually uncertain whether the human orthologue of the identified gene has the same functional role in a real human body.

Genome-wide association study (GWAS) is a powerful tool for dissecting unknown complex traits by identifying loci associated with particular diseases, and the number of GWAS reports has been rapidly increasing. The identified genes or loci could be seeds for functional analysis, risk prediction and personalized medicine. However, the roles of the identified genes in the pathogenesis have typically not been clarified, and further study is required (Hindorff et al. 2009). Another limitation in GWAS is that statistical analysis with only common SNPs may miss some pathological genes for which individual genetic difference cannot be captured with proxy common variants. For example, the power to detect causal rare variants would be too small because of low linkage disequilibrium (LD) between the causal and the proxy common variants. It should be useful to inspect the genes with moderate *p* values while simultaneously looking at other information such as biological pathway, gene expression, and evidence in animal models. Therefore, a translational approach of integrating genetic association study in human and experiments in mouse has a potential value to facilitate finding additional disease-related genes, by taking advantages of both the approaches.

Alzheimer’s disease (AD) is a common neurological disease that causes dementia in humans. Aβ accumulation is the central pathology of Alzheimer’s disease. Molecular pathogenesis of Aβ accumulation for familial AD has been explained by the causative genes, *APP, PSEN1* and *PSEN2* (Hardy and Selkoe 2002; Rogaev et al. 1995; Sherrington et al. 1995). Genetic risk factors have been reported for sporadic AD (*APOE*, etc) (Bertram et al. 2007; Lambert et al. 2013; Saunders and Roses 1993; Saunders et al. 1993a, b; Strittmatter et al. 1993). However, the mechanism, which leads to the accumulation of Aβ in the early stage of the AD, is not well understood (Gaiteri et al. 2016).

Among approaches in the mouse model of human diseases, transcriptome analysis has an advantage: the transcriptome between human and mouse brains is well preserved (Miller et al. 2010), and this may facilitate translational research from mouse to human. APP Tg mice that reproduce Aβ accumulation in brain are widely used as model animals of AD. Taking advantage of transcriptome analysis in the mouse model, our previous study (Gan et al. 2015; Morihara et al. 2014) used a genome-wide transcriptome analysis with various mouse strains with different susceptibilities to Alzheimer’s disease. Genes detected by conventional transcriptome analysis include both causative genes and genes affected by disease pathogenesis. To ensure we detect genes affecting AD pathology, we implemented a two-step approach in our transcriptome analysis. First, we used non-transgenic mice strains with no Alzheimer pathology and selected the genes with differential expression compared to the low-susceptibility strain. This use of non-transgenic mice selects genes for which differences in expression are based on the genetic backgrounds and not secondary effects caused by Aβ accumulation. Second, we used APP transgenic mice with mixed genetic backgrounds to find genes associated with accumulation of Aβ. The top genes whose expression levels were highly correlated with accumulation of Aβ may have roles in the accumulation of Aβ in brain. A further examination of those genes in human or an integrated analysis with human data was desired.

**Fig. 1:**
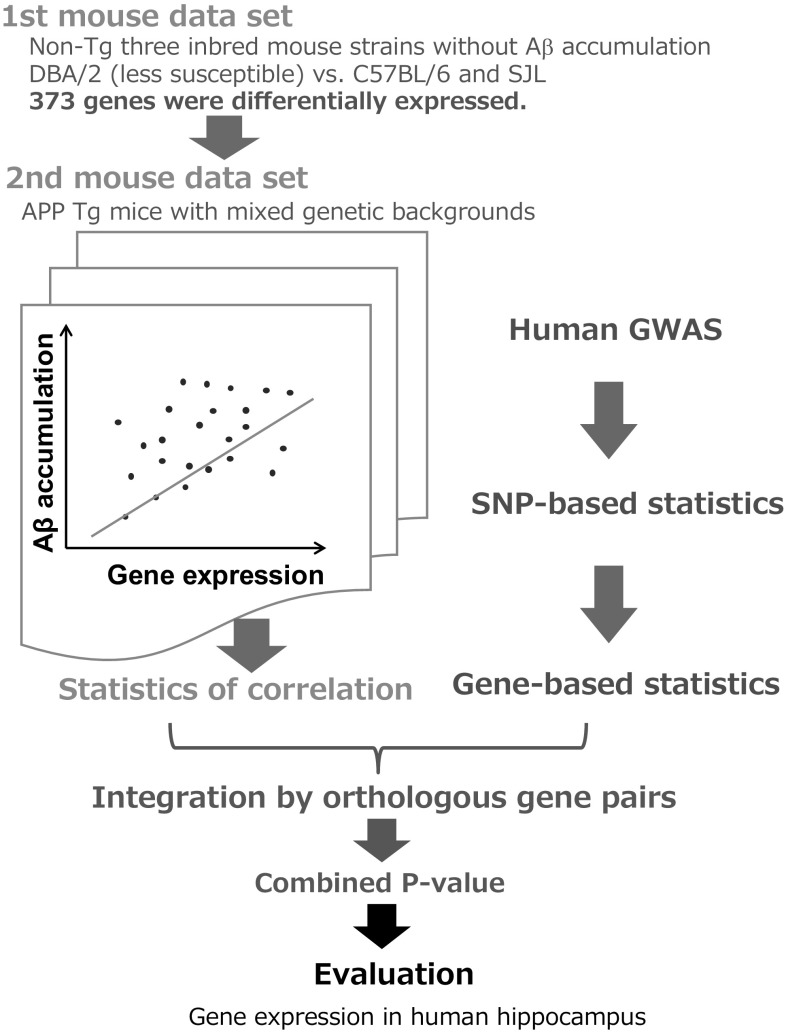
Scheme of integrated analysis of mouse transcriptome and human GWAS. To detect genes affecting AD pathology, we implemented two steps in our transcriptome analysis (green). First, we used non-transgenic mice strains with no Alzheimer pathology and selected the genes with differential expression in the low-susceptibility strain (DBA/2). This use of non-transgenic mice means that differences in gene expression are based on the genetic backgrounds and not secondary effects caused by Aβ accumulation. Second, we used APP transgenic mice with mixed genetic backgrounds to find genes associated with accumulation of Aβ (middle left). In mouse brain, the relationship of Aβ accumulation and gene expression was examined, and *p* value of correlation was obtained. Genome-wide association with AD was conducted with human subjects (Hirano et al. 2015), and SNP-based GWAS statistics were converted into gene-based statistics (blue). Both types of gene-based statistics from mouse and human were integrated through orthologous gene pairs, and a combined *p* value was calculated by the inverse-normal method (also known as Stouffer’s *Z* score method) without weighting (magenta, see “Materials and methods”). Candidate genes were prioritized by the combined *p* values. The significant genes were selected for further evaluation. Human hippocampus postmortem samples were used to determine whether the gene is expressed differently between AD patients and controls

To identify novel AD-related genes that cause Aβ accumulation in the current study, we took an integrated approach by combining statistics from human GWAS and mouse transcriptome experiments (Fig. 1). First, using the correlation between gene expression level and accumulation of Aβ in the mouse model (Morihara et al. 2014), we obtained a *p* value for each mouse gene as the significance of correlation. Second, by utilizing SNP-based statistics in a previous GWAS of human subjects with AD (Hirano et al. 2015), we obtained gene-based statistics from the SNP-based statistics. Third, we combined the results of the two types of analyses using orthologous gene pairs between human and mouse. Then, each gene was evaluated for the susceptibility of AD by the combined *p* value calculated from the two types of *p* values. This integrated analysis detected five significant genes as candidate genes for AD pathogenesis. We examined gene expression level of those genes in human AD subjects, which were independent subjects from the GWAS subjects. Two of the five genes showed lower expression levels with statistical significance in human AD patients than in controls, which is consistent with their mouse orthologues which showed a negative correlation between gene expression level and Aβ accumulation.

## Materials and methods

### Gene expression and Aβ accumulation in transgenic mice

Aβ levels in mouse brains and two sets of genome-wide gene expression data in mouse hippocampus were obtained in a previous study (Morihara et al. 2014) (Fig. 1). The first set of genome-wide gene expression data (12 arrays) was from three inbred non-Transgenic (non-Tg) mouse strains. We choose the genes that were differentially expressed in the mouse strain (DBA/2) with lower susceptibility to AD compared to the other strains (C57BL/6 and SJL). Because these mice carry no APP transgene and have no Aβ pathology, the difference in expression levels is based on their genetic background and not secondary effects caused by Aβ accumulation. From the original transcriptome data containing 13,309 probes for 9964 genes, we selected 373 genes which had significant differential expression (Student’s two-tailed *t* test *p* < 0.001, FDR = 3.05%) in the DBA strain compared to the B6 and SJL strains. These 373 genes reflect physiological changes in neurodegeneration and some may be disease-causing.

The second set of genome-wide gene expression data (28 arrays) was from APP transgenic (Tg) mice with mixed genetic backgrounds from different strains (DBA/2, C57BL/6 and SJL). This APP transgene causes Aβ accumulation in the brain and APP transgenic mice (Tg2576) are widely accepted as AD model animals. The accumulated Aβ levels in these mouse brains were measured by ELISA. Statistical significance of correlation between gene expression levels of the 373 genes selected as above and accumulation of Aβ was tested, and the obtained *p* values were used in the following integrated analysis (see below). All mouse transcriptome datasets used in this study have been deposited in the Gene Expression Omnibus (GEO) database (http://www.ncbi.nlm.nih.gov/geo) under accession GSE40330.

### Gene-based statistics from human GWAS

We used the GWAS statistics of a previous study (Hirano et al. 2015) of 811 AD case individuals and 7504 control individuals with 583,884 autosomal SNPs (Supplementary Fig. 1a). In that study, they used samples belonging to the Hondo cluster (Yamaguchi-Kabata et al. 2008) of the Japanese population, and association analysis was adjusted for age and gender. By checking the distribution of the obtained *p* values (Supplementary Fig. 1b for Q–Q plot; lambda (the genomic inflation factor) = 1.078), any significant confounding effects by ancestry of subjects were not observed. We conducted the principal component analysis with this dataset and obtained principal components (PCs) for their genetic backgrounds. However, we did not include any PCs as covariates for the association analysis, because including them did not reduce lambda (1.076).

From the SNP-based GWAS statistics, gene-based statistics were obtained to conduct the integrated analysis with the other gene-based data. There are several available methods for generating gene-based statistics (Bacanu 2012; Christoforou et al. 2012; Lehne et al. 2011; Li et al. 2011; Liu et al. 2010; Neale and Sham 2004). Basically, they address two issues, (1) the number of SNPs varies among genes and (2) SNPs within the gene are not independent because of local LD. The GATES (gene-based association test using extended Simes procedure) method (Li et al. 2011) is one of these methods to calculate gene-based statistics and is implemented in KGG system (http://grass.cgs.hku.hk/limx/kgg/). This method does not require simulation and the KGG system works with a list of SNP *p* values and LD data. We calculated the gene-based *p* value from the SNP *p* value list using KGG system and LD data using HapMap JPT (Japanese from Tokyo) genotype data. After an examination of how the defined gene regions and LD influence the assignment of SNPs to genes (Supplementary Table 1), each SNP was assigned to a gene (genes) if the SNP is located within the mapped region of the mRNA of the gene including the 3 kb surrounding the 5′ and 3′ flanking regions. In addition, SNPs outside of gene region were assigned to a gene if they were in high LD (*r*^2^ > 0.8) with SNPs within the gene. Using the KGG system, gene-based statistics were obtained for 30,584 transcripts, a set of all human transcripts. On average, 18.0 SNPs were assigned to each gene.

To enroll another type of association study for accumulation of rare and common variants within gene, we used the SKAT_CommonRare function of SKAT (version 1.3.2.1) (Ionita-Laza et al. 2013; Wu et al. 2011) with default parameters for SKAT-C and Burden-C. For each gene, we used the same SNP set described above: SNPs within the gene region including 3 kb upstream and downstream and SNPs under LD with those within the gene region. We included age and gender as covariates for calculating the statistics. Obtained *p* values were integrated with the mouse gene expression *p* values for each gene (see “Integrated analysis”).

### GWAS statistics data from the International Genomics of Alzheimer’s Project (IGAP)

As another dataset for evaluating our methodology, we downloaded GWAS statistics data from the International Genomics of Alzheimer’s Project (IGAP) (Lambert et al. 2013). This GWAS was based on cases and controls of European ancestry. The *p* value list for the combined set of the GWAS (stage 1; 17,008 cases and 37,154 controls) and a follow-up study (stage 2; 8,572 cases and 11,312 controls for 11,632 SNPs after quality-control filtering) was used after gene-based annotation using Annovar (Wang et al. 2010) with the “refGene” table. We selected 954 genes linked to the top SNPs with *P* < 0.001 for further examination. Eleven of these genes were in common with the 373 genes selected from the mouse expression experiment data. For these eleven genes, SNPs in the IGAP stage 1 set were assigned to genes in the same way as described above, and we obtained gene-based statistics using the GATES method implemented in KGG system.

### Integrated analysis

The data of mouse transcriptome and the gene-based statistics from human GWAS were combined using orthologous gene pairs between human and mouse. The orthologous table from Mouse Genome Informatics (http://www.informatics.jax.org) (Shaw 2004) was used to identify orthologous genes between human and mouse. The human and the mouse data were combined for the 373 genes (409 probes for the mouse data). To obtain the combined *p* value for each gene, we used the inverse-normal method (also known as Stouffer’s *z* score method) (Stouffer 1949) without weighting. First, *z* scores for mouse and human *p* values (one tailed) were obtained by the inverse function of standard normal distribution cumulative function, then the averaged *z* score was calculated:

$${Z_{\text{C}}}=\frac{1}{{\sqrt 2 }}\left( {{Z_{{\text{MEXP}}}}+{Z_{{\text{HGWAS}}}}} \right),$$

where *Z*_C_, *Z*_MEXP_, and *Z*_HGWAS_ are *z* scores for combined, mouse expression, and gene-based human statistics of GWAS, respectively. Then, the combined *p* value (one tailed) was obtained by the standard normal distribution with *Z*_C_. Lastly, the combined *p* value was doubled (two tailed). The R programming language (version 3.5.0) was used for this calculation.

### eQTL analysis

We checked whether the SNPs used in this study for each gene were reported eQTLs—SNPs with alleles associated with the expression level of a gene. For this, we used data in GTEx (The Genotype-Tissue Expression (GTEx) project 2013; https://www.gtexportal.org/home/; version 7; Caucasian) (Aguet et al. 2017; GTEx Consortium 2013) for brain tissues: amygdala (*n* = 88), anterior cingulate cortex (BA24; *n* = 109), caudate (basal ganglia; *n* = 144), cerebellar hemisphere (*n* = 125), cerebellum (*n* = 154), cortex (*n* = 136), frontal cortex (BA9; *n* = 118), hippocampus (*n* = 111), hypothalamus (*n* = 108), nucleus accumbens (basal ganglia; *n* = 130), putamen (basal ganglia; *n* = 111), spinal cord cervical (cervical c-1; *n* = 83), and substantia nigra (*n* = 80). To combine data from multiple tissues, we used METASOFT (v2.0.1; http://genetics.cs.ucla.edu/meta/) (Han and Eskin 2011), and looked at *p* values using a fixed effect model and one of the random effect models (Han and Eskin 2011).

### Gene expression in human brain

Tissue samples of human hippocampus were obtained from the brain bank of the Choju Medical Institute of Fukushimura Hospital (Toyohashi, Aichi, Japan), and they were independent of the subjects of the GWAS. Hippocampus samples for AD subjects (*n* = 10) were selected for this gene expression experiment, based on the criteria of the Consortium to Establish a Registry for Alzheimer’s Disease (CERAD) (Fillenbaum et al. 2008; Morris et al. 1989) and Braak stage. Control samples (*n* = 13) were selected from the subjects who had died without dementia. RNA integrity numbers (RIN) of the analyzed human hippocampus tissues were above 7.0 as described previously (Morihara et al. 2014). The protocol used here was approved independently by the local ethics committees of Osaka University and Fukushimura Hospital. Gene expression levels of the significant genes in the integrated analysis were examined in human hippocampus tissues of ten AD patients and 13 control subjects. The levels of mRNA were measured by real-time quantitative polymerase chain reaction (qPCR) assays as previously described (Morihara et al. 2014). The ABI pre-designed qPCR assays were used for GUSB (#Hs00205241-m1), *LBH* (#Hs00368853-m1), *SHF* (#Hs00403125-m1), *C5orf51* (#Hs00420444-m1), and *ARSJ* (#Hs00539912-s1). The expression level of each gene was normalized with that of the *GUSB* gene, because we (Morihara et al. 2014) and others (Miyashita et al. 2014) tested several internal controls and found that GUSB was most stable in human brain. Also, potential covariates were not different between AD and control groups [RIN mean ± SD: 7.88 ± 0.59 (AD) and 8.20 ± 0.63 (control), *p* = 0.21; age mean ± SD: 87.4 ± 6.95 (AD) and 88.7 ± 5.73 (control), *p* = 0.62; and gender (male/female): 3/7 (AD) and 3/11 (control), *p* = 0.67]. Therefore, we applied Student’s *t* test (two-tailed test) with the analysis of difference in average expression levels between the AD and the control groups.

## Results

### Correlation of gene expression and Aβ accumulation level in candidate genes in AD-resistant mouse strain

Using two sets of mouse transcriptome data, we identified 373 candidate genes for regulation of Aβ accumulation in brain, and *p* values of correlation between the levels of expression for each gene and Aβ. Previously, we (Morihara et al. 2014), and others (Jackson et al. 2015; Ryman et al. 2008; Sebastiani et al. 2006), have shown that Aβ accumulation in APP Tg mice with DBA/2 genetic background was significantly lower than those with C57BL/6 and/or SJL. This fact clearly suggests that some genes in DBA/2 suppress Aβ accumulation. To identify these Aβ controlling genes in DBA/2, we first used non-Tg mice. Using non-Tg mice means that any change in gene expression is based on the genetic background and not secondary effects caused by Aβ accumulation. In this study, we selected 373 genes whose expression levels were significantly different (Student’s two-tailed *t* test *p* < 0.001, FDR = 3.05%) in DBA/2 compared with SJL or C57BL/6 (“Materials and methods”) as potential candidate genes controlling Aβ accumulation.

In addition to these three non-Tg inbred mouse strains, we previously prepared APP Tg mice with mixed genetic background of DBA/2 (lower susceptibility to AD), C57BL/6 and SJL (Morihara et al. 2014). We measured the gene expression profile and levels of Aβ in their brains. In this study, we examined the correlation between the expression levels of these 373 genes and Aβ levels in these APP Tg mice. The *p* values of these correlations were used for the subsequent integrated analysis.

### Gene-based statistics

By the conventional approach of genome-wide association study (Hirano et al. 2015) (811 AD case individuals and 7504 control individuals with 583,884 SNPs on autosomes), we observed six significant SNPs with genome-wide significance (*p* < 5.0 × 10^−8^) on 19q13 including the *APOE* gene (Supplementary Fig. 1a), a well-known risk factor of AD (Saunders and Roses 1993; Saunders et al. 1993a, b), and several adjacent genes. In addition to this strong *APOE* signal of chromosome 19, there were also a substantial number of SNPs with moderate *p* values (512 SNPs, *p* < 0.001), which may include additional causative genes for AD.

To conduct gene-based integrated analysis with mouse data, we obtained gene-based statistics from SNP-based GWAS statistics by, first, using GATES method implemented in KGG system (Li et al. 2011) (Table 1; “Materials and methods”). With GWAS alone, we did not observe any significant gene other than *APOE* (2.71 × 10^−19^) and the surrounding genes (*TOMM40* and *PVRL2*), under LD with APOE, although there were additional possible genetic signals of association. Among the 373 candidate genes expressing differently in AD-resistant mouse strain, *ST6GALNAC4, ARRB1, KCNS1, TNNT1, EBNA1BP2, CSRNP3*, and *C5orf51* showed smallest *p* values (Table 1).

**Table 1:** Gene-based statistics from human AD GWAS for the 373 genes

Gene symbol	Gene-based *p* value (GATES)*	Chrom	Genomic start position	Length (bp)	No. of SNPs
*ST6GALNAC4*	0.01188	9	130,661,594	68,411	8
*ARRB1*	0.01375	11	74,891,656	183,314	42
*KCNS1*	0.01444	20	43,588,548	340,988	29
*TNNT1*	0.01498	19	55,620,901	123,172	11
*EBNA1BP2*	0.01776	1	43,497,528	212,692	23
*CSRNP3*	0.01805	2	166,423,989	177,254	54
*C5orf51*	0.01921	5	41,875,954	416,722	20
*ALG14*	0.04124	1	95,434,624	129,468	21
*SLC8A1*	0.04437	2	40,335,143	447,126	154
*SHF*	0.04483	15	45,429,200	135,704	12
*ARSJ*	0.04510	4	114,749,976	164,713	30
*LBH*	0.04624	2	30,430,441	102,618	30
*HPS6*	0.04785	10	103,426,179	438,324	6
*CSRNP1*	0.06052	3	39,149,345	54,295	10
*CP110*	0.06278	16	19,458,391	143,646	9
*HLA-DMA*	0.06749	6	32,901,162	67,186	59
*PHGDH*	0.06765	1	120,212,461	91,943	22
*APPBP2*	0.07607	17	58,313,733	324,939	5
*CNIH4*	0.08004	1	224,377,234	310,930	20
*RSRC1*	0.08348	3	157,649,272	670,528	47
*ADORA1*	0.08498	1	203,054,414	93,835	35
*EML4*	0.08908	2	42,370,747	325,180	51
*ZNF585A*	0.09224	19	37,405,387	265,466	17
*GRIK4*	0.09344	11	120,506,621	359,947	125
*PTPRD*	0.09439	9	8,267,310	2,350,878	1006
*PTPN11*	0.09587	12	111,840,106	1,199,711	34
*RCBTB2*	0.09609	13	48,818,436	312,206	9
*RPS3A*	0.09691	4	151,578,661	526,020	10
*SERPINA3*	0.09790	14	95,058,586	50,822	22

29 genes showing smaller *p* values (*p* < 0.1) are shown

*Gene-based statistics was calculated using GATES method (Li et al. 2011) implemented in KGG system (see “Materials and methods”)

As another independent method of obtaining gene-based statistics from SNP-based GWAS, we also conducted SKAT (Ionita-Laza et al. 2013; Wu et al. 2011) with the option of combining common and low-frequency variants together. Among the 373 candidate genes expressing differently in AD-resistant mouse strain, *BOK, ELOVL4, THAP4, ARRB1, ARSJ, TRIM3*, and *PTPN11* showed smallest *p* values (Supplementary Table 2).

We also used GWAS statistics from the International Genomics of Alzheimer’s Project (IGAP) (Lambert et al. 2009) to evaluate the effectiveness of our approach. We selected 954 genes linked to top SNPs with *p* < 0.001 for examination, 11 of which were in common with the 373 genes selected from the mouse expression experiment data (Supplementary Table 3).

### Integrated analysis

First, to evaluate the feasibility of our methodology, we analyzed IGAP data considering our mouse experiment data. We took the intersection of the two gene sets, 373 genes from the mouse expression analysis and 954 genes that are linked to top SNPs (*p* < 0.001) in the IGAP dataset (1st and 2nd combined), and obtained 11 shared genes. For these genes, we looked at the results from our integrated analysis with GATES (Supplementary Table 3). By combining the mouse expression data, these genes which are top hits in IGAP, obtained much better results, and showed significant/moderate *p* values in our results also. This result supports the validity of our approach. Therefore, we proceeded to the next analysis: integration of our human GWAS and mouse experiment data for the remaining genes.

Next, we took the results from our GATES analysis of the original GWAS (Hirano et al. 2015) dataset and the mouse expression data and obtained a combined *p* value for each gene from the two *p* values (mouse transcriptome analysis and human genetic association) through the inverse-normal method. Five genes showed significant combined *p* values with a significance level of *p* < 0.000067 (= 0.05/373/2): *LBH* (limb bud and heart development), *ST6GALNAC4* (ST6-*N*-acetylgalactosaminide alpha-2,6-sialyltransferase 4), *ARSJ* (arylsulfatase family, member J), *C5orf51*, and *SHF* (Src homology 2 domain-containing F) (Table 2). These five genes had nominal *p* values through GWAS alone (gene-based *p* values ranged from 0.011 to 0.046), and multiple SNPs whose *p* values were very different (Supplementary Table 4; Supplementary Fig. 2). However, they were the top significant genes when human genetic association and mouse transcriptome data were integrated. When we compared our results to the GTEx data, we found that many SNPs, particularly those with p < 0.05 in our human GWAS, are eQTLs linked to *LBH* and *SHF* (Supplementary Table 5). Furthermore, they are more relevant in anterior cingulate cortex BA24, cortex, and frontal cortex BA9 tissues, where Aβ accumulation tends to be observed more frequently than in other tissues.

**Table 2:** Top genes in the integrated analysis

Mouse gene expression and Aβ accumulation				Human GWAS gene-based statistics (GATES)		Combined *p* value
Gene	Cor*	Up/down	*p* val (cor)	Gene	*p* value**	
*Lbh*	− 0.6941	Down	0.000042	*LBH*	0.046239	1.66E−05
*St6galnac4*	0.5970	Up	0.000797	*ST6GALNAC4*	0.011877	3.32E−05
*Arsj*	0.6609	Up	0.000129	*ARSJ*	0.045099	3.73E−05
*AW549877*	− 0.6009	Down	0.000722	*C5orf51*	0.019214	5.20E−05
*Shf*	− 0.6455	Down	0.000208	*SHF*	0.044829	5.31E−05

Data for five genes [*p* < 0.05/(373*2) in the integrated analysis] are shown

See Supplementary Table 4 for SNP-based statistics for the five top genes

*Correlation coefficient of Aβ accumulation and gene expression level

**Gene-based statistics was calculated using GATES method (Li et al. 2011) implemented in KGG system (see “Materials and methods”)

Also, as another integrated analysis approach, we integrated *p* values from our SKAT analysis of the same GWAS and the mouse gene expression analysis for each of the 373 genes (Supplementary Table 6). Nine genes: *ARSJ, ELOVL4, THAP4, EXOC2, KLK8, ATXN1, ARRB1, RPS3*, and *RPAIN* had* p* < 0.000067 (= 0.05/373/2). Note that these genes have *p* < 0.05 for both the human GWAS SKAT and mouse gene expression results. Also, by checking GTEx, we found that most of these genes have multiple eQTLs within them (Supplementary Table 7). Within and surrounding the *ARSJ* gene region, we found multiple promising eQTLs linked to these genes, although none were significant in the GWAS. Most SNPs within and surrounding the *ELOVL4* gene region are, interestingly, promising eQTLs of this gene, although this gene itself does not have GWAS hit SNPs. All significant GWAS SNPs near *THAP4* are also eQTLs of this gene. For *EXOC2*, all nearby significant GWAS SNPs are eQTLs, and most SNPs within and surrounding this gene are, interestingly, very strong eQTLs of this gene. For *KJK8*, we did not observe eQTLs for GWAS hits or SNPs around this gene. The expression level of this gene might be irrelevant in human, or eQTLs might exist outside of the analyzed region. Although the *ATXN1* gene had no significant GWAS SNPs, there are many eQTLs associated with this gene. The *ARRB1* gene has a strong overlap between eQTLs and GWAS SNPs with* p* < 0.05, and *RPS3* and *RPAIN* have several very strong eQTLs, although they did not overlap with the GWAS results.

### Gene expression level in human autopsy subjects

**Fig. 2:**
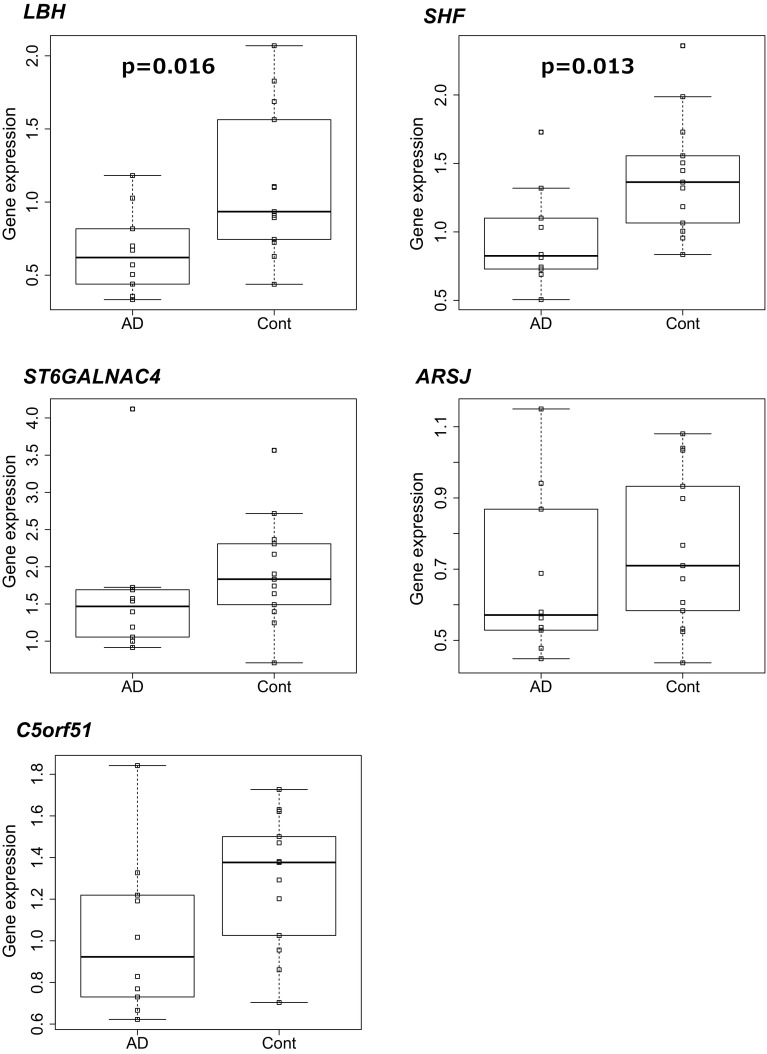
Comparison of gene expression levels in human hippocampus. Gene expression levels for the five significant genes were examined in postmortem human subjects (10 AD patients and 13 control individuals), who were not included in the AD GWAS. Difference in average expression levels between the AD group and the control group was tested with the Student’s *t* test (two-tailed test)

To validate biological roles of the five genes identified by our GATES–GWAS and mouse integrated analysis (*LBH, ST6GALNAC4, ARSJ, C5orf51*, and *SHF*) in human brain, we examined gene expression level of these genes in the hippocampus of AD patients and control autopsy individuals (sample sizes are 13 and 10, respectively), who were independent of the GWAS subjects. Among the five genes tested, gene expression levels of *LBH* and *SHF* were significantly different (FDR < 0.05) (Fig. 2). In both *LBH* and *SHF*, gene expression levels were lower in AD patients than control individuals. This observation was in accordance with the expression levels of these genes, which were negatively correlated with the levels of Aβ accumulation in mouse.

## Discussion

To identify genes that cause Aβ accumulation, we conducted an integrated analysis of human genetic association and mouse transcriptome studies, and our results showed that two genes, *LBH* and *SHF*, are suggested to be novel AD-associated genes. Our results suggested that expression level in *LBH* and *SHF* are negatively associated with Aβ accumulation. Both of *LBH* and *SHF* showed lower expression levels in the human hippocampus of pathologically diagnosed AD patients with confirmed levels of excessive Aβ than those of control individuals (Fig. 2). Also, DBA mouse strain which suppresses Aβ accumulation (Jackson et al. 2015; Morihara et al. 2014; Sebastiani et al. 2006) had higher gene expression levels of both *Lbh* and *Shf* than the other strains. Gene expression levels of both genes in *App* Tg mice with mixed genetic backgrounds were negatively correlated with accumulation of Aβ (Table 2).

*LBH* (limb bud and heart development) is a homolog of mouse *Lbh*, which is a transcription factor and is involved in development of limb bud and heart (Ai et al. 2008; Briegel and Joyner 2001). *LBH* was reported as a direct target of the Wnt signaling pathway (Rieger et al. 2010). Though the mechanisms are still unclear, cross-talk between the Wnt pathway and Alzheimer’s disease has been reported (Inestrosa and Arenas 2010). The levels of Wnt signaling in AD patients are low, suggesting that reduced Wnt signaling could be the triggering factor for Aβ production (Inestrosa and Arenas 2010). From a previous GWAS, human *LBH* has been reported to be associated with autoimmune disease such as rheumatoid arthritis (Okada et al. 2014). A recent study (Ekwall et al. 2015) showed that *LBH* is involved in synovial pathology of rheumatoid arthritis. Interestingly, previously reported AD-associated loci include genes involved in immune systems (Bettens et al. 2013; Gjoneska et al. 2015; Lambert et al. 2013). As *LBH* is also involved in the autoimmune system, there is a possibility that *LBH* has a role in preventing Aβ accumulation through recognition and interaction with other molecules. Furthermore, data of temporal changes of gene expression may also support that *LBH* is associated with AD pathology. By referring to the Human Brain Transcriptome data (Kang et al. 2011), we confirmed a relatively higher *LBH* expression in the fetal stage which gradually decreases with age. Age-related expression change was also observed in blood (Peters et al. 2015), with older individuals showing decreased expression.

*SHF* (SH2 domain-containing adapter protein F) is suspected to play a role in regulating apoptosis in response to PDGF (platelet-derived growth factor) (Lindholm et al. 2000). It is also known that its gene product interacts with anaplastic lymphoma kinase and negatively regulates its downstream signals in neuroblastoma (Takagi et al. 2013). There is a possibility that *SHF* can act in preventing accumulation of Aβ through its ability to regulate phospho-transduction signals. Although there exists an alternative explanation that the lower expression levels of *LBH* and *SHF* are consequences of AD pathogenesis, we think that the former explanation is more likely because the higher expression levels of *LBH* and *SHF* in mouse DBA strain (vs. C57BL/6 and SLJ strains) were not affected by AD pathogenesis. It is still unclear how *LBH* and *SHF* are related to accumulation of Aβ, and further functional studies would clarify the roles of these two genes in regulating Aβ accumulation and pathogenesis of AD.

Furthermore, using SKAT, another way to obtain gene-based statistics, and integration with mouse transcriptome data, we observed several strong candidate genes: *ELOVL4* (elongation of very long chain fatty acids-like 4) was implicated for the biosynthesis of fatty acids in the pathogenesis of inherited macular degeneration (Zhang et al. 2001), severe neurodevelopmental disorder characterized by ichthyosis, spastic quadriplegia, mental retardation (Aldahmesh et al. 2011), spinocerebellar ataxia-34 (SCA34) and erythrokeratodermia (Giroux and Barbeau 1972). *THAP4* (thap domain-containing protein 4) was listed as one of the potential candidates associated with brain voxel through neuroimaging (Stein et al. 2010). *EXOC2* (exocyst complex component 2) was reported for nominal association with AD age of onset modifier genes through a whole-exome study (Velez et al. 2016). For *KLK8* (kallikrein-related peptidase 8), it was previously shown that its mRNA levels in AD hippocampus were significantly higher than in controls (Shimizu-Okabe et al. 2001), and its protease was recently reported as a suggestive factor for increasing the risk for AD specifically in females (Keyvani et al. 2018). *ATXN1* (ataxin 1) was screened for one of the candidates associated with AD through a GWAS and functionally validated its loss of function of increased Aβ-protein levels by potentiating beta-secretase processing of beta-amyloid precursor protein (Zhang et al. 2010). *ARRB1* (arrestin beta 1) was implicated for negative correlation with the apoptosis of neurons during AD development and progression (Guo et al. 2017). Further study would be required to validate these associations.

The result from the integration of IGAP GWAS and mouse experiment data shows the effectiveness of our methodology, i.e., integrating omics data, for prioritizing candidates of disease-related genes. We think that the analyzed common genes with smaller *p* values in the integrated analysis are worth considering for further investigation (e.g., *ACP2, EXOC2*, and *EML4*; Supplementary Table 3). These results would be the first step after obtaining human GWAS results towards clarification of disease mechanisms through combination with mouse experiment results. However, we observed some discrepancy between the results of IGAP and our GWAS. One of the reasons for this may be that our human GWAS appears to have been underpowered, as the study size was relatively small. The results from the IGAP analysis suggest that, if the study size is large enough, more significant genes would show up as candidates through integration with the mouse transcriptome data. In addition, one of the possible reasons for the difference with the IGAP results may be due to inter-ethnic genetic differences. Furthermore, there is a possibility that the differences in the top genes by our approach and that of IGAP are because we utilized mouse transcriptomic data. A large portion of the heritability of AD has not been identified. Human GWAS and mouse transcriptomics could be very different approaches to AD genes. An estimation showed that GWAS, including *APOE* ε4, explains only 28.57% of the heritability of liability (Cuyvers and Sleegers 2016). Moreover, *APOE* ε4, which cannot be involved in mice as all mice have *APOE* ε4, accounts for the large portion of the GWAS heritability. The *APOE* ε3 and ε2 alleles are unique to human.

Our study shows that combining human genetic association study and mouse transcriptome analysis is feasible and can take advantage of the both approaches. In fact, two genes, among five significant genes in the integrated analysis, were significantly supported by experimental study in independent human subjects. This means that this integrated analysis is effective to find disease-associated genes that were not detected in conventional GWAS. Furthermore, a mouse-to-human translational approach, like our study, can identify novel disease-related genes and give insight into their functional roles. Generally, functional role is usually unknown for the associated genes identified solely by conventional GWAS, because complex diseases may have phenotypic variations, and several biological pathways may be involved in the pathogenesis. As we focused on accumulation of Aβ, which is one of the various phenotypes of AD, the identified genes are suspected to be involved in the accumulation mechanism. Our results also showed that genetic signals of association may be localized to regions within a gene [as seen in *LBH* gene, approximate genomic region (GRCh37/hg19) = Chr2:30,500,000, supplementary table 4]. Grouping SNPs based on functional units, or domains, within a gene may be an alternative way for obtaining statistics for each functional unit of a gene.

At present, the strategies and methods of integrated analysis and translational approaches to find genes for complex diseases are not well established, and there are several limitations to this study. The first thing is the use of statistics for integration. Our approach of combining *p* values from mouse gene expression data and human GWAS statistics is simple, and it would be better to consider effect size (with confidence interval) even if the p value is marginal. This is important because low-frequency variants with strong effect sizes are not likely to show significant *p* values. We tried the sequence kernel association test (SKAT) to overcome this point. However, we used *p* values for integration of the gene-based results from the human GWAS and the mouse expression experiment, and further improvement in methodology is desired. Second, there are other limitations that arise from combining human and mouse data. For example, the location and order of genes may differ between human and mouse genomes, except for in well-conserved syntenic genomic regions. Therefore, effects of genetic variants in cis or trans might also differ between the two species. Our approach, based on integration of gene-based statistics from GWAS and gene-expression levels, aims to detect relationships between the gene itself and phenotype, and is not suitable for detecting possible effects on adjacent genes. Furthermore, regulatory variants, which could be located adjacent/distant to a gene, may be hard to be detected by this gene-based approach. Including eQTLs in the integrated analysis would be quite useful to resolve these issues if the sample size of the eQTL data was much larger. Lastly, we conducted this integrated analysis assuming orthologous genes in human and mouse have similar functions. However, interpretation of results may be complicated or difficult for genes with paralogues, like multigene families. Although both strategy and data type may vary among projects, appropriate design of integration and data evaluation and additional experimental evidence would help clarify how the genes affect complex diseases.

As we showed in this study, a gene-based approach is feasible and powerful to integrate various kinds of data. Further improvements to the methodology would contribute to finding additional disease-causing genes not detected by conventional GWAS. Integration of omics data such as metabolome data (Koshiba et al. 2018), biological pathways and epigenetic data (Gjoneska et al. 2015) would be useful for prioritizing disease-related genes. Then, detected genes would have functional insights that are important for developing therapeutic targets.

## Electronic supplementary material

Below is the link to the electronic supplementary material.

Supplementary material 1 (PDF 868 KB)

Supplementary material 2 (PDF 1849 KB)

Supplementary material 3 (PDF 48 KB)

Supplementary material 4 (XLSX 24 KB)

Supplementary material 5 (XLSX 14 KB)

Supplementary material 6 (XLSX 23 KB)

Supplementary material 7 (XLSX 22 KB)

Supplementary material 8 (XLSX 12 KB)

Supplementary material 9 (XLSX 68 KB)
