# Supplementary material for: Integrated analysis of human genetic association study and mouse transcriptome suggests LBH and SHF genes as novel susceptible genes for amyloid-β accumulation in Alzheimer’s disease
Source: Hum Genet. 2018 Jul 13;137(6):521–33. doi: 10.1007/s00439-018-1906-z (PMC6061045; doi:10.1007/s00439-018-1906-z)
Supplement: Supplementary file 1 — Supplementary material 1 (PDF 868 KB) [file 439_2018_1906_MOESM1_ESM.pdf]

"Integrated analysis of human genetic association study and mouse transcriptome suggest *LBH* and *SHF* genes as novel susceptible genes for amyloid-β accumulation in Alzheimer's disease" by Yamaguchi-Kabata, Morihara, Ohara, Ninomiya, Takahashi, Akatsu, Hashizume, Shigemizu, Boroevich, Kubo, Takeda, Tsunoda
Submitted to *Human Genetics*
E-mail: tatsuhiko.tsunoda@riken.jp (RIKEN Center for Integrative Medical Sciences)

a

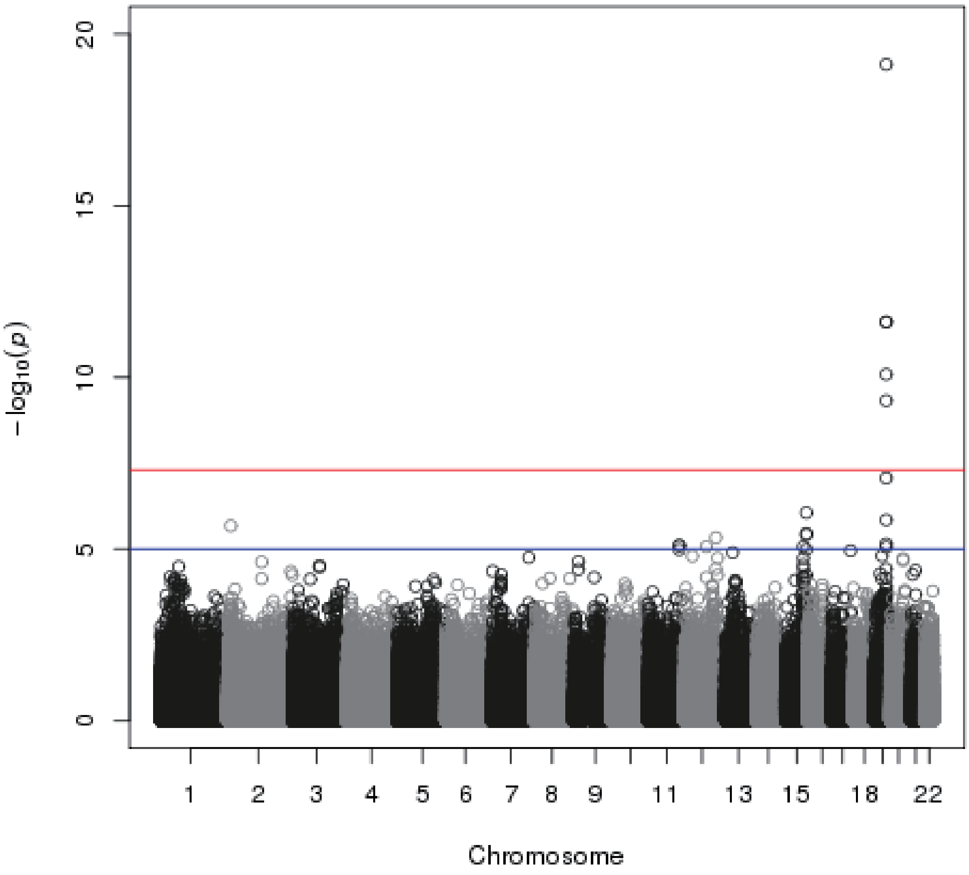

b

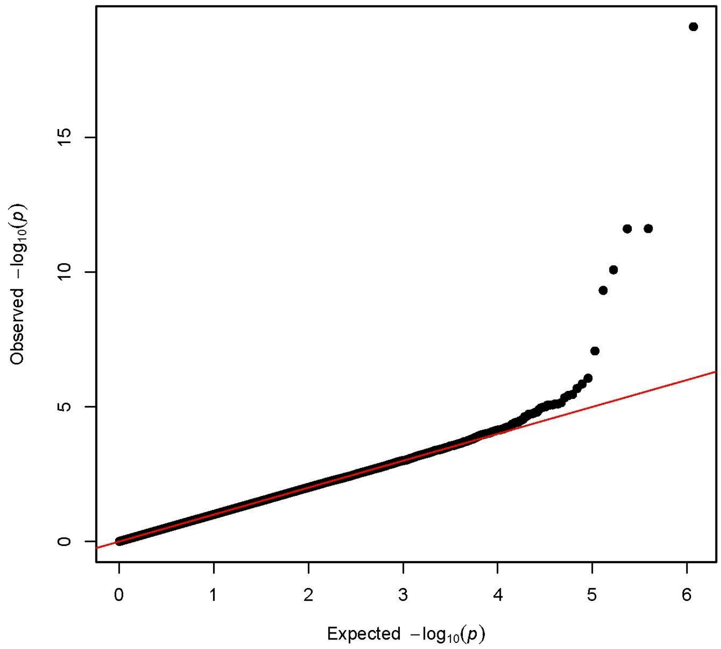

Supplementary Figure 1.
Genome-wide association with Alzheimer' s disease (811 cases and 7504 controls).
Top: A Manhattan plot for p-values for 583,884 SNPs along genomic position. The threshold for genome-wide significance level (p<5x10-8) and suggestive level (P<1x10-5) are indicated by the red line and the blue line.
Bottom: A quantile-quantile plot for P-value distribution.
