# Supplementary material for: Integrated analysis of human genetic association study and mouse transcriptome suggests LBH and SHF genes as novel susceptible genes for amyloid-β accumulation in Alzheimer’s disease
Source: Hum Genet. 2018 Jul 13;137(6):521–33. doi: 10.1007/s00439-018-1906-z (PMC6061045; doi:10.1007/s00439-018-1906-z)

"Integrated analysis of human genetic association study and mouse transcriptome suggest *LBH* and *SHF* genes as novel susceptible genes for amyloid-β accumulation in Alzheimer's disease" by Yamaguchi-Kabata, Morihara, Ohara, Ninomiya, Takahashi, Akatsu, Hashizume, Shigemizu, Boroevich, Kubo, Takeda, Tsunoda
Submitted to *Human Genetics*
E-mail: tatsuhiko.tsunoda@riken.jp (RIKEN Center for Integrative Medical Sciences)

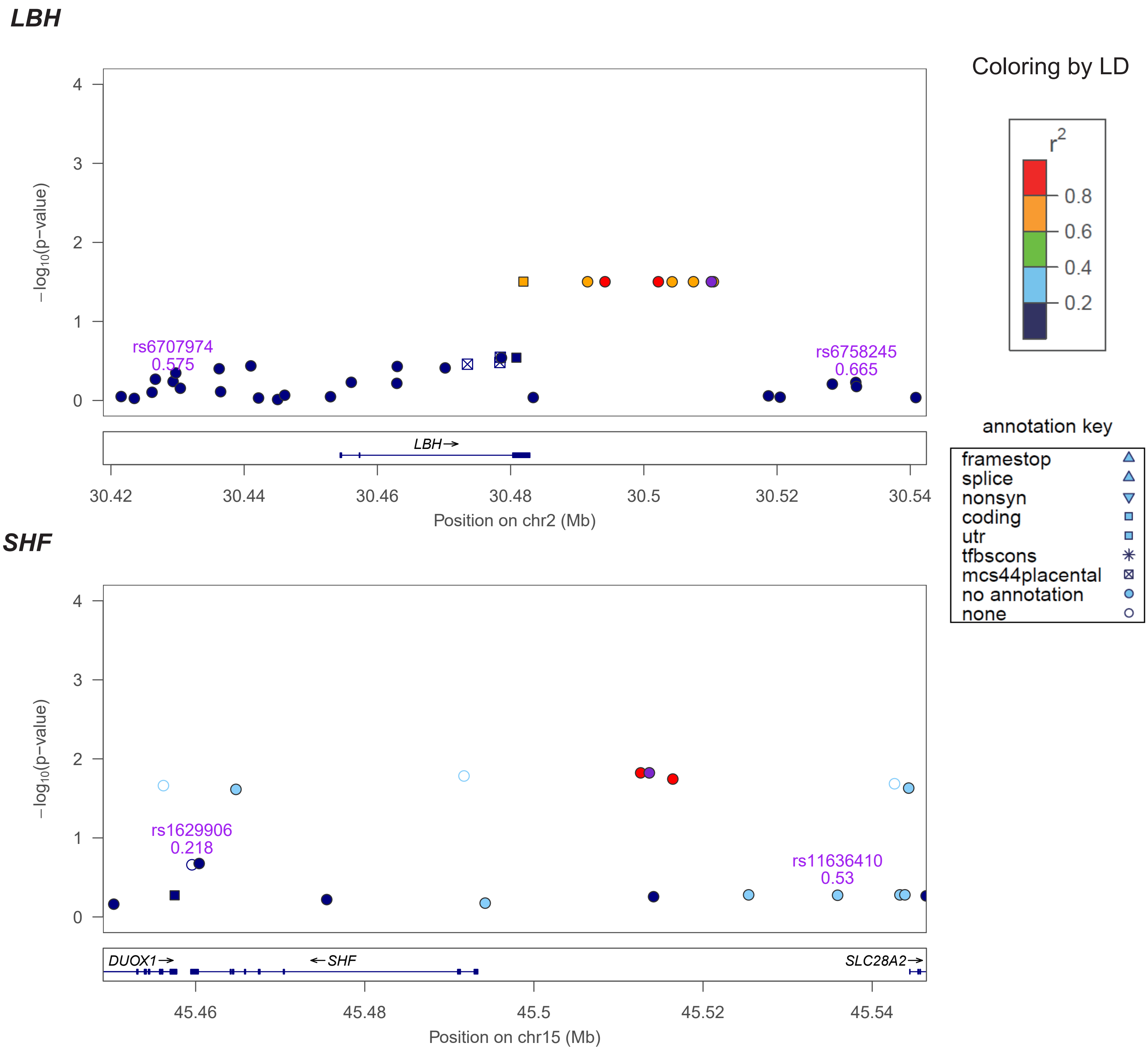

Supplementary Figure 2

Regional plots of GWAS p-values for the five significant genes in the integrated analysis. See supplementary table 2 for details of each SNP. Among the SNPs assigned to the gene, two SNPs at the most 5′ and 3′ ends are labelled. Coloring by linkage disequilibrium (LD) and characters by functional annotations follow a default setting in LocusZoom (Pruim et al. 2010 Bioinformatics) (see right).

Supplementary Figure 2 (continued)

Regional plots of GWAS p-values for the five significant genes in the integrated analysis.

ARSJ

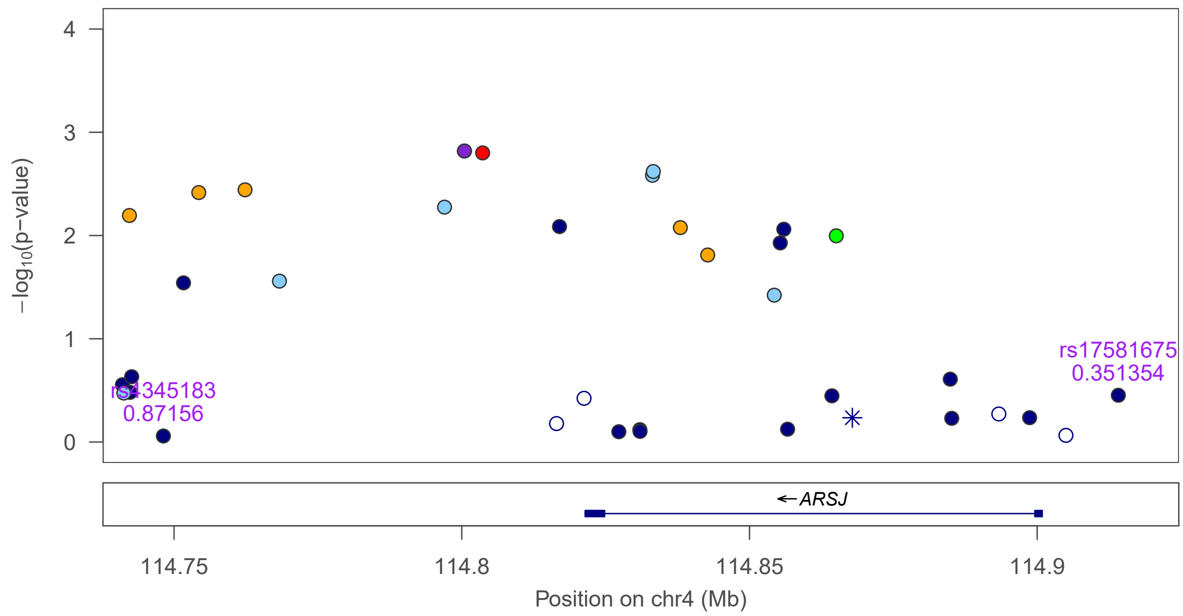

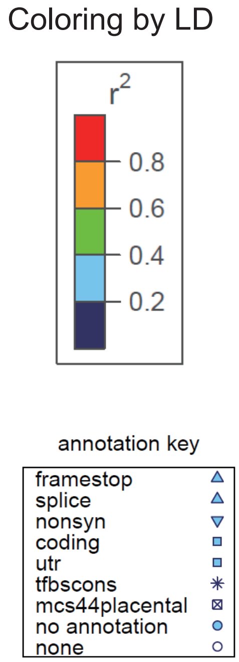

ST6GALNAC4

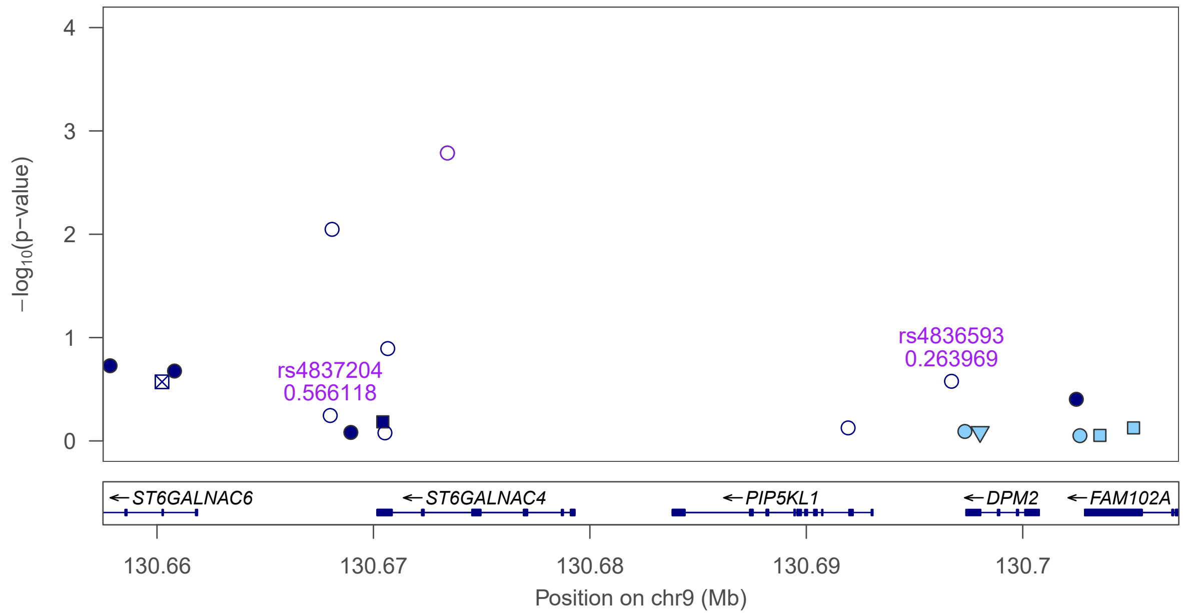

C5orf51

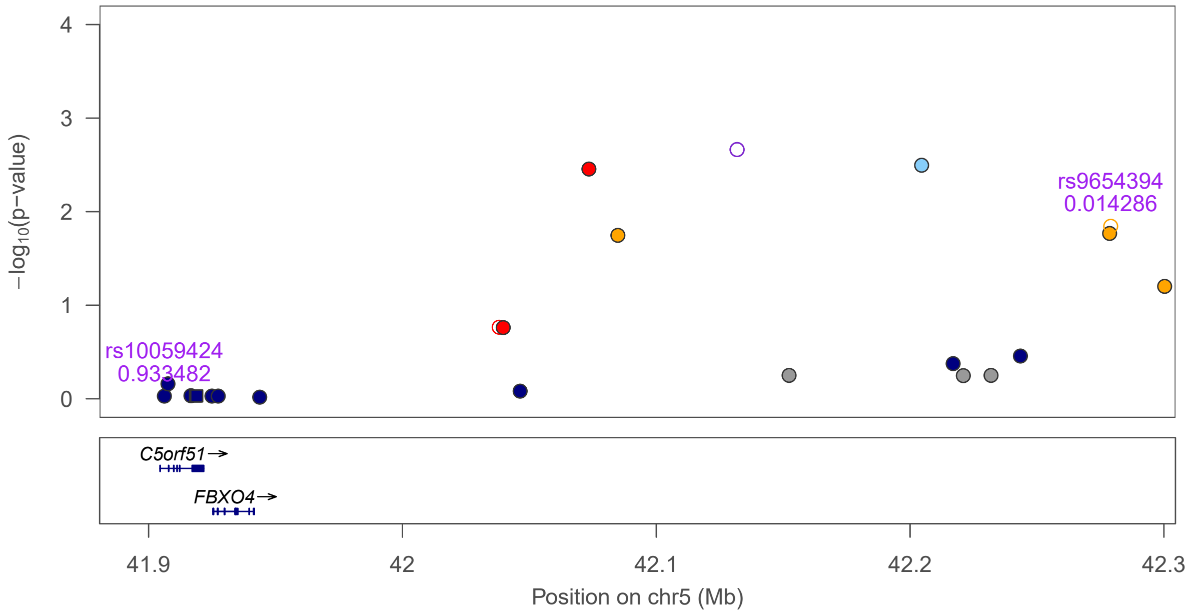

Supplement: Supplementary file 2 — Supplementary material 2 (PDF 1849 KB) [file 439_2018_1906_MOESM2_ESM.pdf]
