# Supplementary material for: Integrated analysis of human genetic association study and mouse transcriptome suggests LBH and SHF genes as novel susceptible genes for amyloid-β accumulation in Alzheimer’s disease
Source: Hum Genet. 2018 Jul 13;137(6):521–33. doi: 10.1007/s00439-018-1906-z (PMC6061045; doi:10.1007/s00439-018-1906-z)
Supplement: Supplementary file 3 — Supplementary material 3 (PDF 48 KB) [file 439_2018_1906_MOESM3_ESM.pdf]

"Integrated analysis of human genetic association study and mouse transcriptome suggest LBH and SHF genes as novel susceptible genes for amyloid-ß accumulation in Alzheimer's disease" by Yamaguchi-Kabata, Morihara, Ohara, Ninomiya, Takahashi, Akatsu, Hashizume, Shigemizu, Boroevich, Kubo, Takeda, Tsunoda E-mail: tatsuhiko.tsunoda@riken.jp (RIKEN Center for Integrative Medical Sciences) Submitted to *Human Genetics*

Suppl. Table 1. Assigning SNPs into genes in different conditions

(A) Use of gene map only

		mRNA region	With 5' and 3' flanking regions		
			3kb flanking	5kb flanking	10kb flanking
Average region length		(65.5 kb)	(71.5 kb)	(75.5 kb)	(85.5 kb)
No. of genes covered by at least one SNP		8233 (92.8%)	8383 (94.5%)	8579 (96.7%)	8872 (100%)
No. of SNPs assigned to any gene		135107 (22.5%)	147223 (24.5%)	153040 (25.5%)	165827 (27.6%)
SNPs assigned to multiple genes		368	3264	5437	11547
	by mRNA	368	368	368	368
	by flanking region	-	2896	5069	11179
No. of SNPs per gene	Mean	15.3	17.0	17.9	20.2
	Median	6	7	8	11
	Range	0 - 1358	0 - 1361	0 - 1363	0 - 1366

Post QC SNPs for autosomes (600,112) were analyzed with the mapped locations for 8872 genes.

(B) SNPs in high LD are also included

		With 3kb flanking regions		With 5kb flanking regions	
		$r^2 >= 0.5$	$r^2 >= 0.8$	$r^2 >= 0.5$	$r^2 >= 0.8$
No. of genes covered by at least one SNP		8800 (99.2%)	8788 (99.1%)	8827 (99.5%)	8820 (99.4%)
No. of SNPs assigned to any gene		202673 (33.8%)	178105 (29.7%)	206801 (34.5%)	182724 (30.4%)
SNPs assigned to multiple genes		42191	25486	45001	28065
	by mRNA	368	368	368	368
	by flanking region	2896	2896	5069	5069
	by use of LD	38927	22222	39564	22628
No. of SNPs per gene	Mean	31.6	24.9	32.9	26.0
	Median	23	16	24	17
	Range	0 - 1363	0 - 1361	0 - 1365	0 -1363

Post QC SNPs for autosomes (600,112) were analyzed with the mapped locations for 8872 genes.
